# 4-Fluoro-2-({[(2*R*)-1-hy­droxy-1,1,3-tri­phenyl­propan-2-yl]imino}­meth­yl)phenol

**DOI:** 10.1107/S2414314620015801

**Published:** 2020-12-11

**Authors:** Fanrui Sha, Adam R. Johnson

**Affiliations:** a Department of Chemistry, Harvey Mudd College, 301 Platt Blvd., Claremont, CA 91711, USA; University of Aberdeen, Scotland

**Keywords:** crystal structure, Schiff base, tridentate ligand, chiral mol­ecule

## Abstract

The title compound, C_28_H_24_FNO_2_, crystallizes in the ortho­rhom­bic space group *P*2_1_2_1_2_1_. A hydrogen-bonding network between the tertiary alcohol group and the fluoro substituent results in [010] chains in the solid state.

## Abstract 2

In the title compound, the salicyl­aldehyde alcohol group is engaged in an intra­molecular O—H⋯N hydrogen bond with the imine nitro­gen atom, while the tertiary alcohol is engaged in a weak inter­molecular O—H⋯F hydrogen bond with an adjacent mol­ecule.

## Graphical abstract

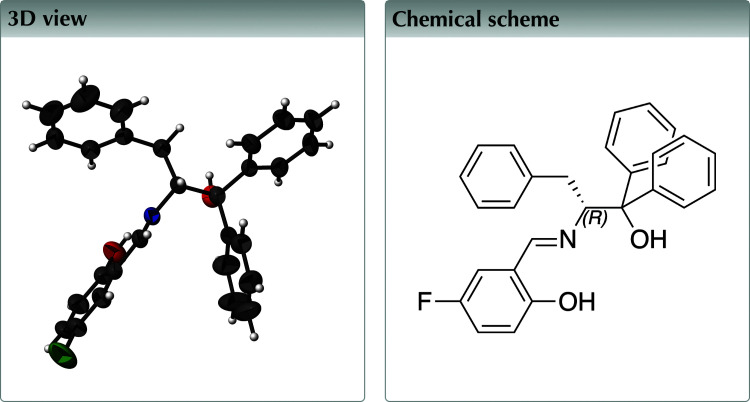

## Structure description

We have synthesized a number of chiral imine diols by Schiff-base condensation of the corresponding salicyl­aldehydes with (*S*)- or (*R*)-2-amino-1,1,3-tri­phenyl­propanol (Kang *et al.*, 2004 ▸; Liu *et al.* 2004 ▸). These compounds serve as ligands for titanium for the asymmetric intra­molecular hydro­amination of amino­allenes (Sha *et al.*, 2019 ▸). We routinely prepare both enanti­omers of the ligands, and a number of them were examined by single-crystal X-ray diffraction, including the l-enanti­omer of the title compound, in order to compare the structures of the free and bound ligand.

**Figure 1:**
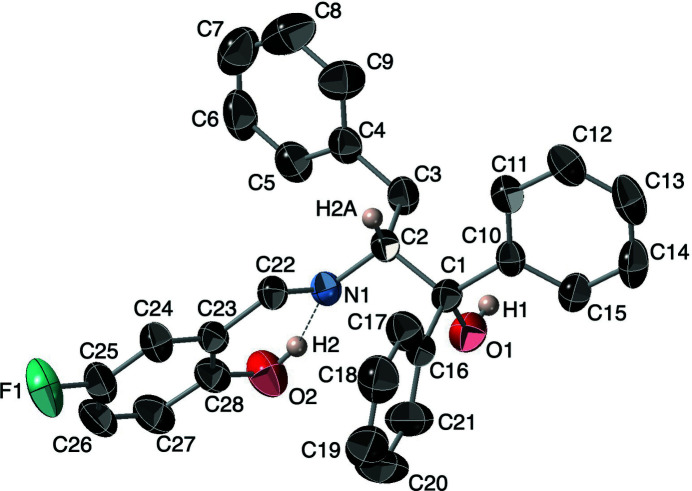
The asymmetric unit of the title compound with displacement ellipsoids shown at the 50% probability level. Hydrogen atoms besides H1, H2 and H2*A* have been omitted for clarity.

2-Hy­droxy-5-fluoro-benzaldehyde 2*S*-(1,1,3-tri­phenyl­propanol) imine, C_28_H_24_FNO_2_, crystallizes in the ortho­rhom­bic space group *P*2_1_2_1_2_1_ as shown in Fig. 1 ▸. The major structural features of the two enanti­omers are similar, as expected. The l-enanti­omer structure was collected at 100 K while the d-enanti­omer was collected at 293 K. The unit-cell parameters in the current room-temperature structure are slightly larger (average 1.3%), presumably due to the higher temperature of the data collection. The absolute structure parameter of −0.1 (3) has a large uncertainty but the absolute configuration was verified by synthesis and polarimetry.

The compound has the expected imine–phenol structure as opposed to the iminium–phenoxide tautomer seen in derivatives with less steric bulk. The C23–C28 phenol aromatic ring is close to co-planar with atoms O2 [deviation from the ring plane = 0.040 (2) Å], C22 [–0.061 (2) Å], N1 [–0.034 (2) Å] and C2 [–0.039 (2) Å]. These four atoms exhibit less deviation from the plane than the enanti­omer. The C22—N1—C2—C1 torsion angle is 110.2 (2)°, which places atom O1 1.555 (2) Å above the plane of the ring. This deviation is 0.166 Å larger than that for the enanti­omer at 100 K, although the torsion angle is almost identical.

The bonds between C27—C28, C23—C28 and C23—C24 are long at 1.39–1.41 Å while those between C24—C25, C25—C26 and C26—C27 are shorter at 1.36–1.37 Å. In contrast, the aromatic rings on the benzyl and phenyl substituents have typical C—C bond distances ranging from 1.37–1.39 Å. The aromatic C28—O2 bond at 1.349 (3) Å is substanti­ally shorter than the aliphatic C1—O1 bond [1.439 (3) Å]. This bonding motif has been seen in related structures (Sha *et al.*, 2019 ▸).

**Figure 2:**
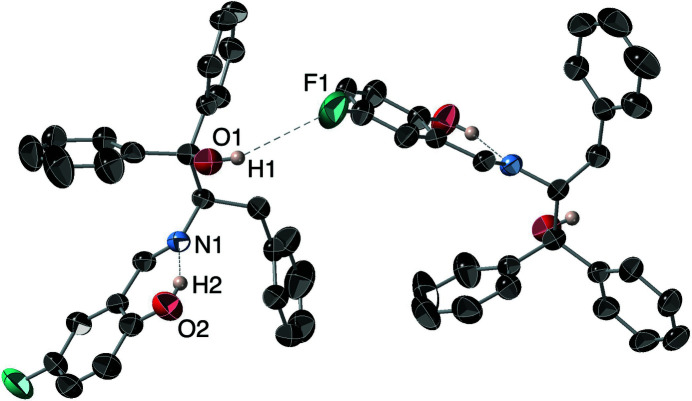
A view of the inter- and intra­molecular hydrogen-bonding network along the *b* axis.

There is an intra­molecular O2—H2⋯N1 hydrogen bond (Table 1 ▸) between the salicyl­aldehyde alcohol group and the imine nitro­gen atom, which closes an *S*(6) ring and a long-range inter­molecular hydrogen bond between the tertiary alcohol O1—H1 and the F1 atom of an adjacent mol­ecule as shown in Fig. 2 ▸: the H⋯F and O⋯F distances are 2.94 and 3.720 (3) Å, respectively. Weak inter­molecular C—H⋯F and C—H⋯O contacts are also observed.

## Synthesis and crystallization

Preparative details of the material have been reported previously (Sha *et al.*, 2019 ▸). Crystals in the form of light-yellow blocks were obtained by slow evaporation from the mixed solvents of hexa­ne/ethyl acetate.

## Refinement

Crystal data, data collection and structure refinement details are summarized in Table 2 ▸.

## Supplementary Material

Crystal structure: contains datablock(s) global, I. DOI: 10.1107/S2414314620015801/hb4369sup1.cif

Structure factors: contains datablock(s) I. DOI: 10.1107/S2414314620015801/hb4369Isup2.hkl

Supporting information file. DOI: 10.1107/S2414314620015801/hb4369Isup3.cml

CCDC reference: 1970566

Additional supporting information:  crystallographic information; 3D view; checkCIF report

## Figures and Tables

**Table 1:** Hydrogen-bond geometry (Å, °)

*D*—H⋯*A*	*D*—H	H⋯*A*	*D*⋯*A*	*D*—H⋯*A*
O2—H2⋯N1	0.82	1.86	2.583 (3)	147
O1—H1⋯F1^i^	0.82	2.94	3.720 (3)	160
C9—H9⋯F1^ii^	0.93	2.54	3.467 (3)	175
C14—H14⋯O2^iii^	0.93	2.58	3.369 (3)	142

Symmetry codes: (i) 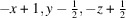 ; (ii) 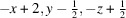 ; (iii) 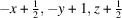 .

**Table 2:** Experimental details

Crystal data	
Chemical formula	C_28_H_24_FNO_2_
*M*_r_	425.48
Crystal system, space group	Orthorhombic, *P*2_1_2_1_2_1_
Temperature (K)	293
*a*, *b*, *c* (Å)	6.0147 (2), 18.8172 (4), 20.4530 (5)
*V* (Å^3^)	2314.87 (11)
*Z*	4
Radiation type	Mo *K*α
μ (mm^−1^)	0.08
Crystal size (mm)	0.29 × 0.27 × 0.23

Data collection	
Diffractometer	Rigaku XtaLAB Mini II
Absorption correction	Analytical [*CrysAlis PRO* (Rigaku OD, 2019 ▸); *ABSPACK* (Rigaku OD, 2017 ▸)]
*T*_min_, *T*_max_	0.995, 0.996
No. of measured, independent and observed [*I* > 2σ(*I*)] reflections	73250, 5721, 4529
*R*_int_	0.044
(sin θ/λ)_max_ (Å^−1^)	0.667

Refinement	
*R*[*F* ^2^ > 2σ(*F* ^2^)], *wR*(*F* ^2^), *S*	0.045, 0.098, 1.03
No. of reflections	5721
No. of parameters	291
H-atom treatment	H-atom parameters constrained
Δρ_max_, Δρ_min_ (e Å^−3^)	0.14, −0.15
Absolute structure	Flack *x* determined using 1550 quotients [(*I* ^+^)−(*I* ^−^)]/[(*I* ^+^)+(*I* ^−^)] (Parsons *et al.*, 2013 ▸)
Absolute structure parameter	−0.1 (3)

Computer programs: *CrysAlis PRO* (Rigaku OD, 2019 ▸), *SHELXS* (Sheldrick, 2008 ▸), *SHELXL* (Sheldrick, 2015 ▸), *OLEX2* (Dolomanov *et al.*, 2009 ▸) and *CrystalMaker* (Palmer, 2020 ▸).

## full crystallographic data

### Crystal data

**Table:**

C_28_H_24_FNO_2_	*D*_x_ = 1.221 Mg m^−^^3^
*M**_r_* = 425.48	Mo *K*α radiation, λ = 0.71073 Å
Orthorhombic, *P*2_1_2_1_2_1_	Cell parameters from 20629 reflections
*a* = 6.0147 (2) Å	θ = 2.0–22.8°
*b* = 18.8172 (4) Å	µ = 0.08 mm^−^^1^
*c* = 20.4530 (5) Å	*T* = 293 K
*V* = 2314.87 (11) Å^3^	Block, clear light yellow
*Z* = 4	0.29 × 0.27 × 0.23 mm
*F*(000) = 896	

### Data collection

**Table:**

Rigaku XtaLAB Mini II diffractometer	5721 independent reflections
Radiation source: fine-focus sealed X-ray tube, Rigaku (Mo) X-ray Source	4529 reflections with *I* > 2σ(*I*)
Graphite monochromator	*R*_int_ = 0.044
Detector resolution: 10.0000 pixels mm^-1^	θ_max_ = 28.3°, θ_min_ = 2.0°
ω scans	*h* = −8→8
Absorption correction: analytical [CrysAlisPro (Rigaku OD, 2019); *ABSPACK* (Rigaku OD, 2017)]	*k* = −25→25
*T*_min_ = 0.995, *T*_max_ = 0.996	*l* = −27→27
73250 measured reflections	

### Refinement

**Table:**

Refinement on *F*^2^	Hydrogen site location: inferred from neighbouring sites
Least-squares matrix: full	H-atom parameters constrained
*R*[*F*^2^ > 2σ(*F*^2^)] = 0.045	*w* = 1/[σ^2^(*F*_o_^2^) + (0.0408*P*)^2^ + 0.2813*P*] where *P* = (*F*_o_^2^ + 2*F*_c_^2^)/3
*wR*(*F*^2^) = 0.098	(Δ/σ)_max_ < 0.001
*S* = 1.03	Δρ_max_ = 0.14 e Å^−^^3^
5721 reflections	Δρ_min_ = −0.15 e Å^−^^3^
291 parameters	Absolute structure: Flack *x* determined using 1550 quotients [(*I*^+^)-(*I*^-^)]/[(*I*^+^)+(*I*^-^)] (Parsons *et al.*, 2013)
0 restraints	Absolute structure parameter: −0.1 (3)
Primary atom site location: structure-invariant direct methods	

### Special details

**Table:**

Geometry. All esds (except the esd in the dihedral angle between two l.s. planes) are estimated using the full covariance matrix. The cell esds are taken into account individually in the estimation of esds in distances, angles and torsion angles; correlations between esds in cell parameters are only used when they are defined by crystal symmetry. An approximate (isotropic) treatment of cell esds is used for estimating esds involving l.s. planes.

### Fractional atomic coordinates and isotropic or equivalent isotropic displacement parameters (Å^2^)

**Table:**

	*x*	*y*	*z*	*U*_iso_*/*U*_eq_	
F1	0.9178 (3)	0.82714 (9)	0.12710 (9)	0.0962 (6)	
O1	0.2298 (2)	0.51725 (9)	0.34826 (8)	0.0552 (4)	
H1	0.203611	0.474846	0.343181	0.083*	
O2	0.2834 (3)	0.62611 (10)	0.17459 (9)	0.0684 (5)	
H2	0.328661	0.596034	0.200364	0.103*	
N1	0.5550 (3)	0.56695 (9)	0.25587 (8)	0.0413 (4)	
C1	0.4599 (3)	0.52649 (11)	0.36563 (10)	0.0398 (4)	
C2	0.6059 (3)	0.51204 (10)	0.30374 (9)	0.0386 (4)	
H2A	0.763405	0.515018	0.315703	0.046*	
C3	0.5589 (4)	0.43882 (11)	0.27290 (10)	0.0505 (5)	
H3A	0.581351	0.402322	0.305740	0.061*	
H3B	0.404359	0.436968	0.259445	0.061*	
C4	0.7040 (4)	0.42272 (10)	0.21473 (10)	0.0488 (5)	
C5	0.6403 (5)	0.44232 (13)	0.15197 (12)	0.0639 (7)	
H5	0.505460	0.465438	0.145449	0.077*	
C6	0.7771 (7)	0.42755 (14)	0.09884 (13)	0.0835 (10)	
H6	0.733148	0.441041	0.057053	0.100*	
C7	0.9761 (7)	0.39330 (17)	0.10751 (18)	0.0886 (11)	
H7	1.067032	0.383652	0.071824	0.106*	
C8	1.0393 (6)	0.37362 (18)	0.1685 (2)	0.0909 (10)	
H8	1.173814	0.350153	0.174553	0.109*	
C9	0.9049 (5)	0.38825 (15)	0.22170 (14)	0.0712 (7)	
H9	0.951195	0.374498	0.263194	0.085*	
C10	0.5143 (3)	0.47796 (10)	0.42366 (9)	0.0402 (4)	
C11	0.7181 (4)	0.44477 (12)	0.43239 (11)	0.0495 (5)	
H11	0.827905	0.449448	0.400675	0.059*	
C12	0.7598 (5)	0.40449 (12)	0.48822 (11)	0.0594 (6)	
H12	0.896877	0.382316	0.493428	0.071*	
C13	0.5999 (5)	0.39739 (13)	0.53549 (12)	0.0657 (7)	
H13	0.628493	0.370747	0.572854	0.079*	
C14	0.3965 (5)	0.42993 (14)	0.52737 (11)	0.0652 (7)	
H14	0.287022	0.424937	0.559155	0.078*	
C15	0.3550 (4)	0.46988 (12)	0.47220 (11)	0.0520 (6)	
H15	0.217525	0.491873	0.467401	0.062*	
C16	0.4910 (4)	0.60386 (11)	0.38657 (10)	0.0450 (5)	
C17	0.6909 (5)	0.62610 (12)	0.41351 (12)	0.0576 (6)	
H17	0.803348	0.593111	0.420758	0.069*	
C18	0.7254 (6)	0.69653 (14)	0.42975 (14)	0.0764 (8)	
H18	0.860574	0.710554	0.447662	0.092*	
C19	0.5618 (7)	0.74562 (15)	0.41960 (19)	0.0930 (11)	
H19	0.584347	0.792885	0.431100	0.112*	
C20	0.3641 (7)	0.72472 (17)	0.3923 (2)	0.1065 (13)	
H20	0.253617	0.758201	0.384393	0.128*	
C21	0.3279 (5)	0.65428 (15)	0.37652 (17)	0.0797 (9)	
H21	0.191920	0.640668	0.358886	0.096*	
C22	0.6980 (4)	0.61408 (10)	0.24233 (9)	0.0405 (4)	
H22	0.837032	0.612055	0.262127	0.049*	
C23	0.6501 (4)	0.67165 (10)	0.19634 (10)	0.0415 (5)	
C24	0.8092 (4)	0.72373 (12)	0.18420 (11)	0.0534 (6)	
H24	0.944724	0.723285	0.206112	0.064*	
C25	0.7621 (5)	0.77581 (12)	0.13918 (11)	0.0600 (6)	
C26	0.5670 (5)	0.77790 (13)	0.10519 (12)	0.0649 (7)	
H26	0.542130	0.813151	0.074170	0.078*	
C27	0.4081 (5)	0.72738 (13)	0.11734 (12)	0.0643 (7)	
H27	0.274328	0.728610	0.094574	0.077*	
C28	0.4451 (4)	0.67427 (11)	0.16341 (11)	0.0494 (5)	

### Atomic displacement parameters (Å^2^)

**Table:**

	*U*^11^	*U*^22^	*U*^33^	*U*^12^	*U*^13^	*U*^23^
F1	0.1196 (15)	0.0738 (10)	0.0954 (12)	−0.0342 (10)	−0.0053 (12)	0.0365 (9)
O1	0.0371 (8)	0.0709 (10)	0.0577 (9)	−0.0053 (8)	−0.0042 (7)	0.0022 (8)
O2	0.0602 (11)	0.0715 (11)	0.0735 (12)	−0.0102 (9)	−0.0239 (9)	0.0191 (9)
N1	0.0450 (10)	0.0434 (9)	0.0355 (8)	−0.0008 (8)	−0.0026 (7)	0.0031 (7)
C1	0.0344 (10)	0.0472 (11)	0.0379 (10)	−0.0011 (9)	−0.0006 (9)	0.0030 (8)
C2	0.0407 (11)	0.0398 (10)	0.0354 (9)	−0.0013 (8)	0.0003 (9)	0.0055 (8)
C3	0.0633 (15)	0.0433 (11)	0.0447 (11)	−0.0084 (11)	0.0065 (11)	0.0011 (9)
C4	0.0647 (15)	0.0364 (10)	0.0451 (12)	−0.0073 (10)	0.0022 (11)	−0.0033 (8)
C5	0.0920 (19)	0.0495 (12)	0.0503 (14)	0.0007 (14)	0.0024 (14)	0.0016 (11)
C6	0.144 (3)	0.0599 (15)	0.0463 (14)	−0.013 (2)	0.0211 (18)	0.0026 (11)
C7	0.108 (3)	0.0727 (19)	0.085 (2)	−0.018 (2)	0.041 (2)	−0.0203 (17)
C8	0.073 (2)	0.090 (2)	0.109 (3)	0.0033 (18)	0.013 (2)	−0.035 (2)
C9	0.0765 (19)	0.0720 (16)	0.0652 (16)	0.0083 (15)	−0.0036 (15)	−0.0167 (13)
C10	0.0460 (12)	0.0388 (10)	0.0358 (10)	−0.0093 (9)	−0.0003 (9)	0.0010 (8)
C11	0.0504 (13)	0.0506 (11)	0.0476 (12)	−0.0049 (11)	−0.0025 (10)	0.0081 (9)
C12	0.0697 (17)	0.0526 (13)	0.0557 (14)	−0.0031 (12)	−0.0173 (14)	0.0105 (11)
C13	0.099 (2)	0.0578 (14)	0.0406 (12)	−0.0197 (15)	−0.0153 (14)	0.0125 (11)
C14	0.089 (2)	0.0683 (15)	0.0383 (12)	−0.0223 (15)	0.0110 (13)	0.0044 (11)
C15	0.0564 (14)	0.0538 (12)	0.0458 (12)	−0.0077 (11)	0.0086 (11)	−0.0009 (10)
C16	0.0505 (13)	0.0439 (11)	0.0408 (10)	0.0046 (10)	0.0119 (10)	0.0044 (9)
C17	0.0706 (17)	0.0487 (12)	0.0537 (13)	−0.0050 (12)	−0.0038 (12)	−0.0010 (10)
C18	0.098 (2)	0.0607 (15)	0.0702 (17)	−0.0204 (17)	0.0091 (17)	−0.0113 (13)
C19	0.122 (3)	0.0468 (15)	0.110 (3)	−0.0052 (19)	0.050 (2)	−0.0141 (16)
C20	0.103 (3)	0.0553 (17)	0.162 (4)	0.0271 (19)	0.033 (3)	0.001 (2)
C21	0.0665 (19)	0.0636 (16)	0.109 (2)	0.0174 (15)	0.0101 (17)	−0.0004 (16)
C22	0.0433 (11)	0.0440 (10)	0.0342 (9)	−0.0007 (9)	−0.0013 (9)	0.0033 (8)
C23	0.0537 (13)	0.0385 (10)	0.0323 (9)	0.0028 (9)	0.0016 (9)	0.0003 (8)
C24	0.0629 (15)	0.0514 (12)	0.0458 (12)	−0.0055 (11)	−0.0026 (11)	0.0075 (10)
C25	0.0844 (18)	0.0432 (12)	0.0522 (13)	−0.0071 (12)	0.0050 (14)	0.0087 (10)
C26	0.096 (2)	0.0462 (12)	0.0527 (14)	0.0142 (14)	−0.0048 (15)	0.0117 (10)
C27	0.0745 (18)	0.0602 (14)	0.0581 (14)	0.0113 (14)	−0.0164 (14)	0.0101 (12)
C28	0.0578 (14)	0.0465 (11)	0.0439 (11)	0.0045 (11)	−0.0071 (11)	0.0011 (9)

### Geometric parameters (Å, º)

**Table:**

F1—C25	1.368 (3)	C12—H12	0.9300
O1—H1	0.8200	C12—C13	1.370 (4)
O1—C1	1.439 (3)	C13—H13	0.9300
O2—H2	0.8200	C13—C14	1.378 (4)
O2—C28	1.349 (3)	C14—H14	0.9300
N1—C2	1.456 (2)	C14—C15	1.379 (3)
N1—C22	1.266 (3)	C15—H15	0.9300
C1—C2	1.564 (3)	C16—C17	1.387 (3)
C1—C10	1.533 (3)	C16—C21	1.380 (3)
C1—C16	1.529 (3)	C17—H17	0.9300
C2—H2A	0.9800	C17—C18	1.382 (3)
C2—C3	1.541 (3)	C18—H18	0.9300
C3—H3A	0.9700	C18—C19	1.365 (5)
C3—H3B	0.9700	C19—H19	0.9300
C3—C4	1.506 (3)	C19—C20	1.371 (5)
C4—C5	1.389 (3)	C20—H20	0.9300
C4—C9	1.379 (4)	C20—C21	1.382 (5)
C5—H5	0.9300	C21—H21	0.9300
C5—C6	1.391 (4)	C22—H22	0.9300
C6—H6	0.9300	C22—C23	1.463 (3)
C6—C7	1.371 (5)	C23—C24	1.392 (3)
C7—H7	0.9300	C23—C28	1.406 (3)
C7—C8	1.355 (5)	C24—H24	0.9300
C8—H8	0.9300	C24—C25	1.374 (3)
C8—C9	1.384 (4)	C25—C26	1.364 (4)
C9—H9	0.9300	C26—H26	0.9300
C10—C11	1.387 (3)	C26—C27	1.371 (4)
C10—C15	1.388 (3)	C27—H27	0.9300
C11—H11	0.9300	C27—C28	1.391 (3)
C11—C12	1.393 (3)		
			
C1—O1—H1	109.5	C12—C13—H13	120.2
C28—O2—H2	109.5	C12—C13—C14	119.7 (2)
C22—N1—C2	120.07 (17)	C14—C13—H13	120.2
O1—C1—C2	108.61 (16)	C13—C14—H14	120.0
O1—C1—C10	108.93 (16)	C13—C14—C15	120.1 (2)
O1—C1—C16	107.55 (17)	C15—C14—H14	120.0
C10—C1—C2	113.77 (16)	C10—C15—H15	119.3
C16—C1—C2	108.88 (15)	C14—C15—C10	121.4 (2)
C16—C1—C10	108.92 (16)	C14—C15—H15	119.3
N1—C2—C1	107.61 (15)	C17—C16—C1	120.29 (19)
N1—C2—H2A	109.3	C21—C16—C1	121.7 (2)
N1—C2—C3	108.70 (16)	C21—C16—C17	117.9 (2)
C1—C2—H2A	109.3	C16—C17—H17	119.5
C3—C2—C1	112.55 (16)	C18—C17—C16	121.0 (3)
C3—C2—H2A	109.3	C18—C17—H17	119.5
C2—C3—H3A	108.9	C17—C18—H18	119.9
C2—C3—H3B	108.9	C19—C18—C17	120.3 (3)
H3A—C3—H3B	107.7	C19—C18—H18	119.9
C4—C3—C2	113.37 (18)	C18—C19—H19	120.2
C4—C3—H3A	108.9	C18—C19—C20	119.5 (3)
C4—C3—H3B	108.9	C20—C19—H19	120.2
C5—C4—C3	121.1 (2)	C19—C20—H20	119.8
C9—C4—C3	121.4 (2)	C19—C20—C21	120.5 (3)
C9—C4—C5	117.5 (2)	C21—C20—H20	119.8
C4—C5—H5	119.8	C16—C21—C20	120.9 (3)
C4—C5—C6	120.4 (3)	C16—C21—H21	119.6
C6—C5—H5	119.8	C20—C21—H21	119.6
C5—C6—H6	119.7	N1—C22—H22	119.2
C7—C6—C5	120.6 (3)	N1—C22—C23	121.70 (19)
C7—C6—H6	119.7	C23—C22—H22	119.2
C6—C7—H7	120.2	C24—C23—C22	120.0 (2)
C8—C7—C6	119.5 (3)	C24—C23—C28	119.53 (19)
C8—C7—H7	120.2	C28—C23—C22	120.43 (19)
C7—C8—H8	119.8	C23—C24—H24	120.7
C7—C8—C9	120.4 (3)	C25—C24—C23	118.6 (2)
C9—C8—H8	119.8	C25—C24—H24	120.7
C4—C9—C8	121.6 (3)	F1—C25—C24	118.9 (3)
C4—C9—H9	119.2	C26—C25—F1	118.5 (2)
C8—C9—H9	119.2	C26—C25—C24	122.6 (2)
C11—C10—C1	123.82 (18)	C25—C26—H26	120.4
C11—C10—C15	117.93 (19)	C25—C26—C27	119.2 (2)
C15—C10—C1	118.15 (19)	C27—C26—H26	120.4
C10—C11—H11	119.7	C26—C27—H27	119.7
C10—C11—C12	120.6 (2)	C26—C27—C28	120.6 (2)
C12—C11—H11	119.7	C28—C27—H27	119.7
C11—C12—H12	119.8	O2—C28—C23	121.85 (19)
C13—C12—C11	120.3 (3)	O2—C28—C27	118.8 (2)
C13—C12—H12	119.8	C27—C28—C23	119.3 (2)

### Hydrogen-bond geometry (Å, º)

**Table:**

*D*—H···*A*	*D*—H	H···*A*	*D*···*A*	*D*—H···*A*
O2—H2···N1	0.82	1.86	2.583 (3)	147
O1—H1···F1^i^	0.82	2.94	3.720 (3)	160
C9—H9···F1^ii^	0.93	2.54	3.467 (3)	175
C14—H14···O2^iii^	0.93	2.58	3.369 (3)	142

Symmetry codes: (i) −*x*+1, *y*−1/2, −*z*+1/2; (ii) −*x*+2, *y*−1/2, −*z*+1/2; (iii) −*x*+1/2, −*y*+1, *z*+1/2.
